# Synaptic and functional linkages between spinal premotor interneurons and hand-muscle activity during precision grip

**DOI:** 10.3389/fncom.2013.00040

**Published:** 2013-04-25

**Authors:** Tomohiko Takei, Kazuhiko Seki

**Affiliations:** ^1^Department of Neurophysiology, National Institute of NeuroscienceTokyo, Japan; ^2^Department of Developmental Physiology, National Institute for Physiological SciencesOkazaki, Japan; ^3^PRESTO, Japan Science and Technology AgencyTokyo, Japan

**Keywords:** muscle synergy, grasping, spinal cord, spike-triggered average, cross-correlation

## Abstract

Grasping is a highly complex movement that requires the coordination of a number of hand joints and muscles. Previous studies showed that spinal premotor interneurons (PreM-INs) in the primate cervical spinal cord have divergent synaptic effects on hand motoneurons and that they might contribute to hand-muscle synergies. However, the extent to which these PreM-IN synaptic connections functionally contribute to modulating hand-muscle activity is not clear. In this paper, we explored the contribution of spinal PreM-INs to hand-muscle activation by quantifying the synaptic linkage (SL) and functional linkage (FL) of the PreM-INs with hand-muscle activities. The activity of 23 PreM-INs was recorded from the cervical spinal cord (C6–T1), with EMG signals measured simultaneously from hand and arm muscles in two macaque monkeys performing a precision grip task. Spike-triggered averages (STAs) of rectified EMGs were compiled for 456 neuron–muscle pairs; 63 pairs showed significant post-spike effects (PSEs; i.e., SL). Conversely, 231 of 456 pairs showed significant cross-correlations between the IN firing rate and rectified EMG (i.e., FL). Importantly, a greater proportion of the neuron–muscle pairs with SL showed FL (43/63 pairs, 68%) compared with the pairs without SL (203/393, 52%), and the presence of SL was significantly associated with that of FL. However, a significant number of pairs had SL without FL (SL∩!FL, *n* = 20) or FL without SL (!SL∩FL, *n* = 203), and the proportions of these incongruities exceeded the number expected by chance. These results suggested that spinal PreM-INs function to significantly modulate hand-muscle activity during precision grip, but the contribution of other neural structures is also needed to recruit an adequate combination of hand-muscle motoneurons.

## Introduction

Grasping is a highly complex movement that requires the coordination of a number of hand joints and muscles. The large number of degrees of freedom (DOF) of hand anatomy enable its flexible and varied movement, but this requires a high computational load and causes the “DOF problem” (Wing et al., 1996). Previous electromyographic (EMG) studies in non-human primates showed that hand-muscle activity can be explained by a linear combination of a few basic components (i.e., muscle synergy), suggesting that the neural system reduces hand anatomy DOF by using muscle synergies as modules (Brochier et al., 2004; Overduin et al., 2008).

Neural implementation of muscle synergy has been extensively investigated for hind-limb movement in frogs (Giszter et al., 1993; Mussa-Ivaldi et al., 1994; Tresch et al., 1999; Saltiel et al., 2001; Bizzi et al., 2002) and rats (Tresch and Bizzi, 1999), and spinal interneuron involvement has been suggested. As for hand-muscle synergy, we showed that spinal premotor interneurons (PreM-INs) had divergent facilitatory effects in multiple finger muscles by compiling the spike-triggered averages (STAs) of rectified EMGs in monkeys performing a precision grip task (Takei and Seki, 2010), and PreM-INs showed significant trial-to-trial correlations with grip force and target muscle activity (Takei and Seki, 2006). These results suggested that PreM-IN divergent connections facilitate the coactivation of hand muscles and that they could contribute to hand-muscle synergy formation. However, the extent to which these PreM-IN synaptic connections functionally contribute to activate hand muscles and to build muscle synergy is not clear.

In this study, we specifically tested how PreM-IN output contributes to hand-muscle activation by quantifying PreM-IN synaptic linkage (SL) and functional linkage (FL) with hand-muscle activity (Miller et al., 1993; McKiernan et al., 2000; Holdefer and Miller, 2002). In two macaque monkeys performing a precision grip task, we recorded PreM-IN activity in the cervical spinal cord (C6–T1), with EMG signals measured simultaneously from hand and arm muscles. SL was quantified by testing the existence of post-spike effects (PSEs) with STAs of the rectified EMG. FL was determined by calculating the long-term cross-correlation between the PreM-IN firing rate and each rectified EMG. Then, we compared the SL and FL and examined the associations between the presence of SL and of FL. Our results showed that SL and FL between PreM-IN and their target muscle activities were significantly associated, indicating that spinal PreM-INs significantly contribute to modulating hand-muscle activity involved in grasp control. However, a significant number of incongruities between SL and FL were also found, suggesting that other neural structures contributed to recruiting an adequate combination of hand-muscle motoneurons.

## Materials and methods

### Animals

Electrophysiological recordings were obtained from two adult macaque monkeys (*monkey A*: *Macaca fuscata*, male, 6.8 kg, and *monkey E*: *Macaca mulatta*, male, 5.6 kg). Experiments were performed in accordance with the National Institutes of Health Guidelines for the Care and Use of Laboratory Animals and were approved by the Animal Research Committee at the National Institute for Physiological Sciences, Japan.

### Precision grip task

**Figure 1:**
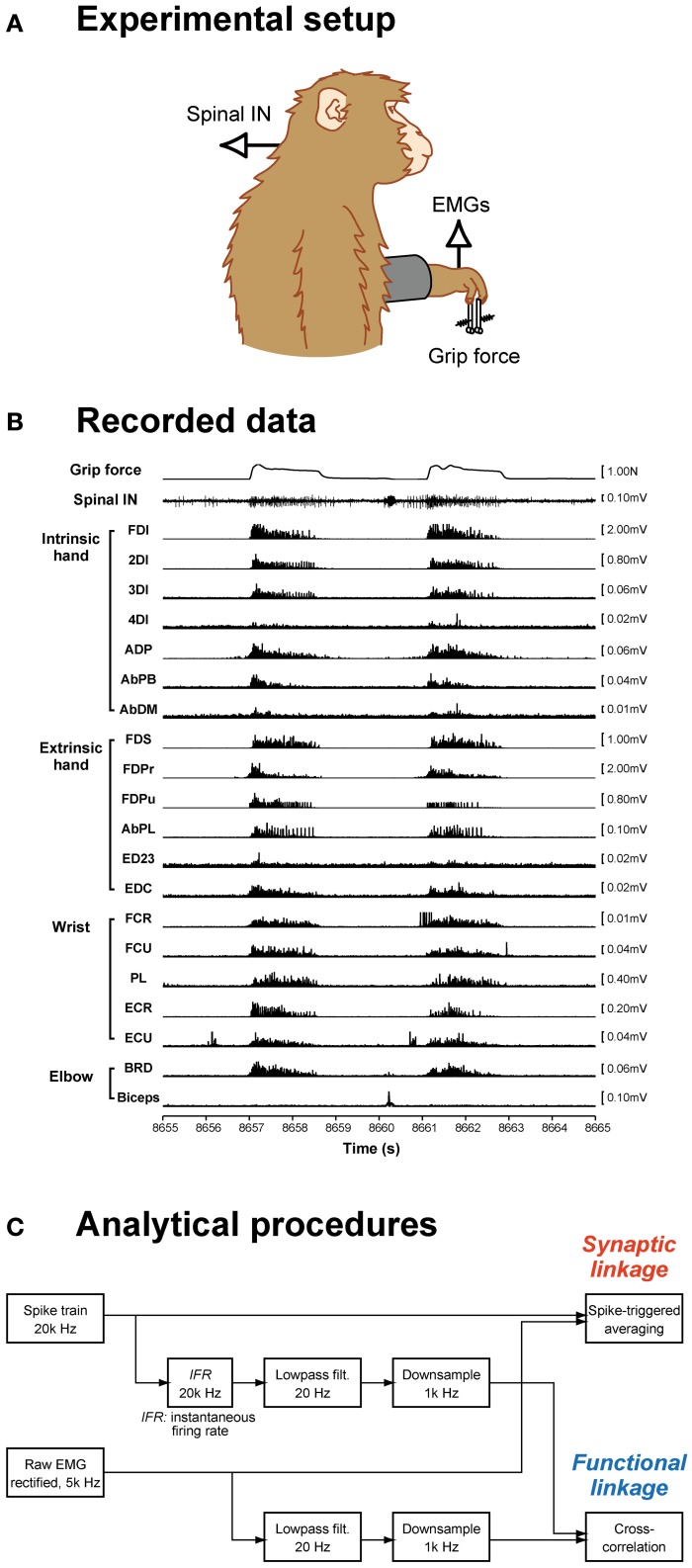
**Data recording and analysis procedures. (A)** Spinal interneuron (IN) activity and forelimb EMG activities were recorded while monkeys performed a precision grip task. **(B)** The signals recorded during two successive trials are shown: grip force (*top*), spinal interneuron firing (*middle*), and 20 EMG recordings (*bottom*). **(C)** Data analysis procedures. Spike-triggered averages were compiled using a spike train and rectified EMG signals (*top*). Cross-correlations were calculated from the neural signal, which was transformed to an instantaneous firing rate signal, low-pass filtered, and downsampled, and EMG signals, which were low-pass filtered and downsampled (*bottom*).

Details of the behavioral task, surgical operations, experimental setup, and procedures for recording single-unit and EMG activity were described previously (Takei and Seki, 2008, 2010). Briefly, monkeys were trained to grip spring-loaded levers with the index finger and thumb (precision grip task, Figure 1A). The lever positions were displayed on a computer screen as cursors, and monkeys were required to track targets. Each trial consisted of a rest period (1.0–2.0 s), lever grip, lever hold (1.0–2.0 s), and lever release. Successful completion of a trial was rewarded with a drop of applesauce. The force required to reach the target positions was adjusted individually for the index finger and thumb (monkey A: 0.4–2.0 N for index finger, 1.0–3.0 N for thumb; monkey E: 0.6–1.1 N for index finger, 0.1–0.3 N for thumb).

### Surgical procedures and data acquisition

Unilateral laminectomy of vertebrae C5–T1 was performed while the animals were anesthetized with isoflurane (1.0–2.0% in 2:1 O_2_:N_2_O) or sevoflurane (1.5–3.0% in 2:1 O_2_:N_2_O) under aseptic conditions, and a custom-made recording chamber was implanted over the laminectomy (Perlmutter et al., 1998). During the recording, the monkey was seated in a primate chair with the head and upper back restrained. Single-unit activities from C5–T1 were recorded with a Tungsten or Elgiloy microelectrode. EMGs from the hand, forearm, and upper arm muscles were simultaneously recorded (Figure 1B). For EMG recording, pairs of stainless steel wires (AS632, Cooner Wire) were chronically implanted subcutaneously in 19 (monkey A) or 20 (monkey E) forelimb muscles, including intrinsic hand muscles: first, second, third, and fourth dorsal interosseous (FDI, 2DI, 3DI, and 4DI, respectively); adductor pollicis (ADP); abductor pollicis brevis (AbPB); abductor digiti minimi (AbDM); extrinsic hand muscles: flexor digitorum superficialis (FDS), radial and ulnar parts of flexor digitorum profundus (FDPr and FDPu), abductor pollicis longus (AbPL), extensor digitorum-2,3 (ED23), extensor digitorum-4,5 (ED45), extensor digitorum communis (EDC); wrist muscles: flexor carpi radialis (FCR), flexor carpi ulnaris (FCU), palmaris longus (PL), extensor carpi radialis longus and brevis (ECRl and ECRb), extensor carpi ulnaris (ECU); and elbow muscles: brachioradialis (BRD), pronator teres (PT), biceps brachii (biceps), and triceps brachii (triceps). The muscles recorded in each monkey were tabulated in a previous paper (Takei and Seki, 2010). Data recorded over at least 10 trials for each single unit were included in the present dataset.

### Spike-triggered averaging of rectified EMGs

To quantify the SL from spinal INs to hand motoneuron pools, we computed the STA of rectified EMGs (Figure 1C). Details of the STA method were described previously (Takei and Seki, 2010). Briefly, STAs were compiled off-line for neuron–muscle pairs with at least 2000 recorded action potentials. All spikes recorded during whole-task phases (i.e., rest, grip, hold, and release phases and intertrial intervals) were used to compile the STAs. EMG was rectified and averaged over an interval of 80 ms, beginning 30 ms before and ending 50 ms after the spike onset. The baseline STA trend was subtracted using the incremented-shifted averaging (ISA) method (Davidson et al., 2007), and then the STA was smoothed with a flat five-point finite impulse response filter. Significant STA effects were identified with multiple-fragment statistical analysis (Poliakov and Schieber, 1998) at *p* < 0.0025 (*p* < 0.05 after Bonferroni's correction). The test window was set at a duration of 12 ms (i.e., between 3 and 15 ms) after the spinal neuron spike. Potential cross-talk between simultaneously recorded EMGs was evaluated by combining a cross-correlation method (Buys et al., 1986) and the third EMG differentiation (Kilner et al., 2002). STA effects potentially resulting from cross-talk between EMG recordings were eliminated from the present dataset.

The STAs of rectified EMGs can produce two types of effects: PSEs and synchrony effects (Schieber and Rivlis, 2005). PSEs reflect the mono- or disynaptic effects of trigger neurons on the motoneuron pool that facilitate or suppress the EMG signal (Fetz and Cheney, 1980). In contrast, synchrony effects are derived from synaptic inputs from other neurons in the motoneuron pool that are synchronized with the discharges of the trigger neurons (Fetz and Cheney, 1980; Schieber and Rivlis, 2005). Therefore, synchrony effects can appear in STAs even if no mono- or disynaptic connection exists between the trigger neuron and the motoneuron pool. Based on criteria established by Schieber and Rivlis (2005), we discriminated PSEs from other synchrony effects according to the onset latency and peak width at half maximum (PWHM) of the STA effects (Schieber and Rivlis, 2005). Onset latency was defined as the time when the averaged EMG exceeded two standard deviations (SDs) from the baseline mean (from 10 to 30 ms before the trigger). PWHM of the STA effect was determined by finding the level that was half of the peak amplitude above (or below for a trough) the baseline mean and by measuring the width of the peak (or trough) at this level. The earliest possible onset latency of the PSEs was set at 3.5 ms based on our previous investigation (Takei and Seki, 2010). The largest PSE PWHM was set at 7 ms based on theoretical considerations (Baker and Lemon, 1998). Therefore, if a neuron produced PSEs with an onset latency of >3.5 ms and PWHM of <7 ms on at least one muscle, the neuron was identified as a PreM-IN.

### Cross-correlation between PreM-IN and EMG activity

To quantify the FL between neuronal activity and hand-muscle activity, we calculated cross-correlations between the neuronal and muscle activities (Figure 1C). First, the instantaneous firing rate [*IFR*(*t*)] of PreM-INs was calculated as the inverse of the interspike interval:

$$IFR(t)=\frac{1}{t_{i\;+\;1}-t_{i}},\text{ for }t_{i}<t<t_{i\;+\;1},$$

where *t*_*i*_ is the time of the *i*th spike. The instantaneous firing rate was then low-pass filtered (second order, Butterworth, cutoff of 20 Hz in forward and backward directions) and down-sampled to 1000 Hz. Rectified EMGs were also low-pass filtered (second order, Butterworth, cutoff of 20 Hz in forward and backward directions) and down-sampled to 1000 Hz. Continuously recorded 90-s data points, which contained ~10 successive trials including whole-task phases, were used to calculate the cross-correlation. Cross-correlation significance was defined by a Monte Carlo method (Miller et al., 1993). To obtain the cross-correlations between non-correlated signals, we transposed the first and second halves of the spinal IN rate signal and calculated the full set of the cross-correlations to obtain the distribution of the peak values between the uncorrelated signals. The 0.5th and 99.5th percentiles of this distribution were used as the lower and upper levels of significance for the cross-correlations (i.e., *p* < 0.01). The transposed signal cross-correlations were compiled for 456 neuron–muscle pairs, and the lower and upper limits were set at −0.29 and 0.25, respectively. These analyses were performed off-line using MATLAB (MathWorks).

## Results

### Synaptic linkage between spinal PreM-INs and hand-muscle activity

**Figure 2:**
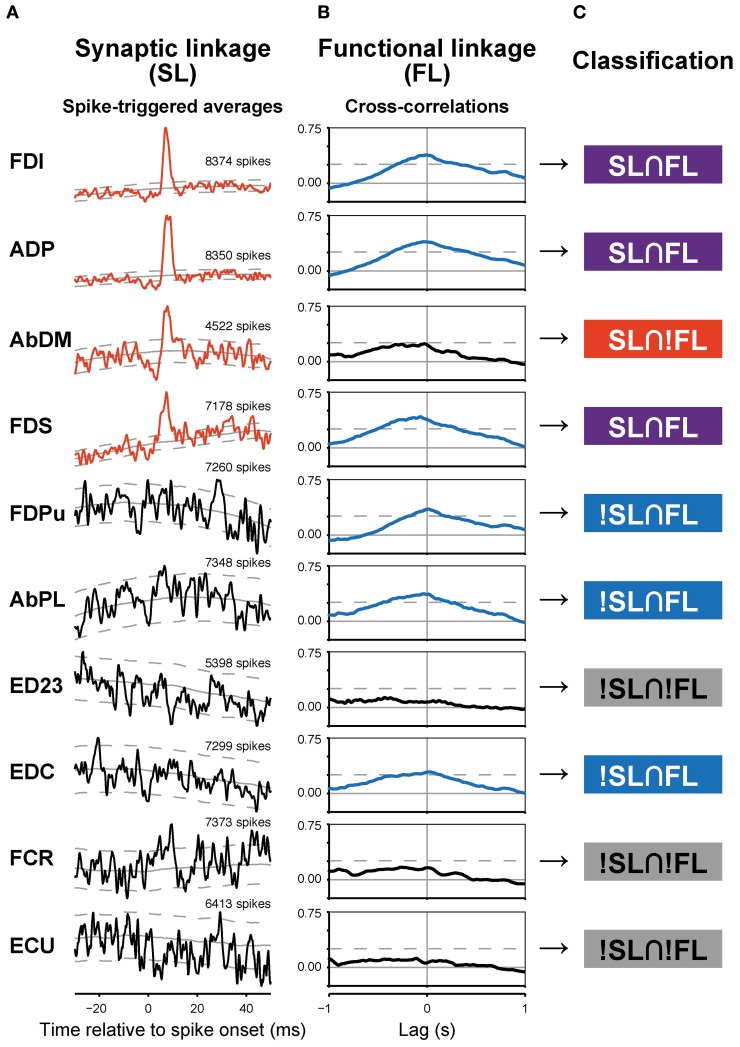
**Synaptic (SL) and functional linkages (FL) between a PreM-IN and hand muscles. (A,B)** Spike-triggered averages **(A)** and cross-correlations **(B)** from a single PreM-IN (Monkey E, T1 segment). Data from 10 muscles were selected. Spike-triggered averages with significant post-spike effects (PSEs) are shown in red, and cross-correlations with significant peaks are shown in blue. The solid and dashed gray lines indicate the background level and significance limits, respectively. **(C)** Each neuron–muscle pair was categorized into one of four groups according to the existence of significant SL and FL as follows: both SL and FL were significant (SL∩FL); SL was significant but not FL, or vice versa (SL∩!FL and !SL∩FL); and neither SL nor FL was significant (!SL∩!FL).

Among the 210 spinal neurons recorded from the two monkeys (34 in monkey A, 176 in monkey E), 23 neurons produced 63 significant PSEs (51 facilitations and 12 suppressions, SL) in hand and arm muscles, and were identified as PreM-INs (18 excitatory and five inhibitory). The neurons had either post-spike facilitation (PSF) or post-spike suppression (PSS) effects on at least one muscle; no neuron had both PSF and PSS simultaneously. As an example, a single PreM-IN STA is shown in Figure 2A. This IN produced significant PSF in four hand muscles (FDI, ADP, AbDM, and FDS). In total, PreM-INs produced PSE in 2.7 ± 2.1 [mean ± standard deviation (SD)] muscles (excitatory: 2.8 ± 2.1; inhibitory: 2.4 ± 2.1) on average, which is referred to as a muscle field (Fetz and Cheney, 1980; Buys et al., 1986). This result indicated that the spinal PreM-INs had a divergent hand-muscle field rather than affecting the activity of a single muscle.

### Functional linkage between spinal PreM-INs and hand-muscle activity

A majority of PreM-INs (19 of 23; 83%), including 17 excitatory and two inhibitory PreM-INs, had significant cross-correlations with at least one muscle (FL). In total, 246 of 456 neuron–muscle pairs had significant cross-correlations. Interestingly, FL polarity was positively biased; most FLs were positive (231 of 246; 94%), and only a few pairs showed negative FLs (15 of 246; 6%). Moreover, all PreM-INs with significant FLs had positive FLs regardless of whether they were excitatory or inhibitory PreM-INs; two excitatory PreM-INs concurrently had negative FLs (Table 1). This result suggested that the excitatory and inhibitory PreM-INs were mostly coactivated with hand muscles during precision grip rather than being reciprocally activated. An example of cross-correlations in a single PreM-IN (same neuron as shown in **A**) is shown in Figure 2B. This IN had a significant positive cross-correlation with six hand muscles (FDI, ADP, FDS, FDPu, AbPL, and EDC). In total, PreM-INs had a FL with 10.7 ± 6.7 muscles on average, and the size was significantly larger than that of the muscle field of SL (*p* < 0.05, *t*-test). This result indicates that PreM-IN activity had significant covariation with muscles other than those on which they had output effects.

**Table 1:** **Summary of FLs of excitatory and inhibitory PreM-INs**.

**Functional linkage**					
**PreM-IN**	**Positive only**	**Negative only**	**Both**	**No FL**	**Total (cells)**
Excitatory	15	0	2	1	18
Inhibitory	2	0	0	3	5
Total	17	0	2	4	23

### Association between synaptic and functional linkages

To test the relationship between synaptic connections and functional covariation further, SL and FL pairwise association was tested. The example in Figure 2 shows various combinations of SL and FL. For example, FDI showed both significant PSF and cross-correlation, indicating that the PreM-IN had a strong excitatory effect on the motoneurons of this muscle, and their activities strongly covaried. This implies a causal relationship, as the PreM-IN activity modulated the target muscle activity. In addition to these congruent cases, however, there were many incongruent instances. The AbDM had a clear significant PSF from the PreM-IN, but the cross-correlation of their activities was not significant. In another example, FDPu showed a clear cross-correlation peak, but it had no significant PSE on the STA. To quantify the association between SL and FL, the neuron–muscle pairs were categorized into four groups according to the existence of significant SL and FL (Figure 2C): both SL and FL were significant (SL∩FL), SL was significant but FL was not, or vice versa (SL∩!FL and !SL∩FL); and neither SL nor FL was significant (!SL∩!FL). In the PreM-INs shown in Figure 2, three pairs (pairs with FDI, ADP, FDS) were SL∩FL, one pair (AbDM) was SL∩!FL, three pairs (FDPu, AbPL, and EDC) were !SL∩FL, and three pairs (ED23, FCR, and ECU) were !SL∩!FL.

**Figure 3:**
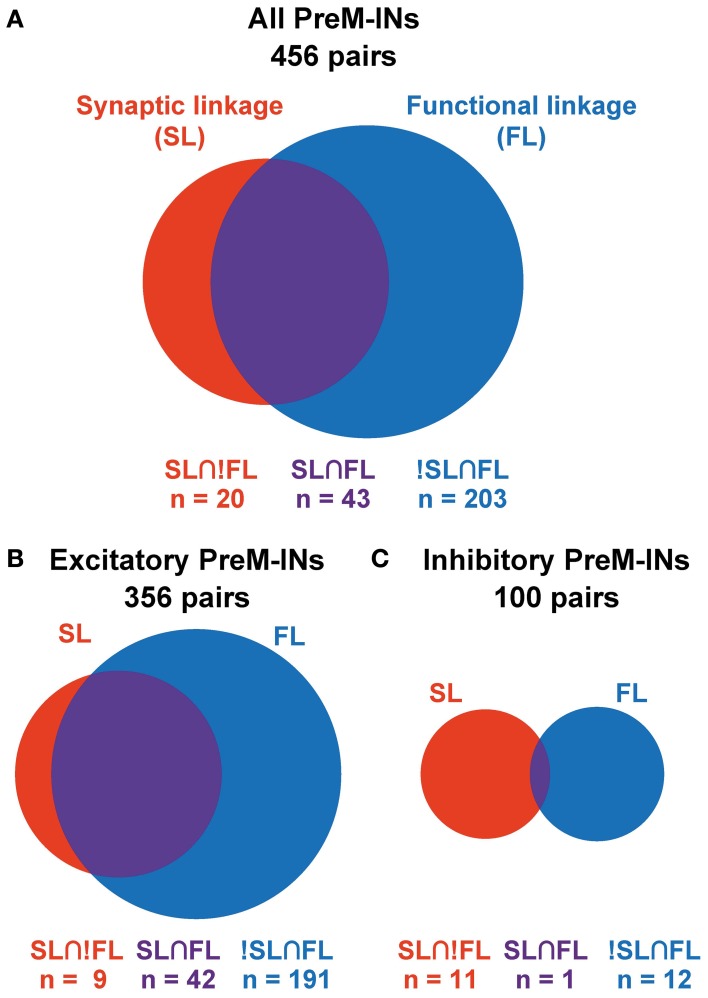
**Association between SL and FL.** Venn diagrams showing the association between SL and FL in all PreM-INs **(A)**, excitatory PreM-INs **(B),** and inhibitory PreM-INs **(C)**. Relative size of the red, blue, and purple areas are proportional to the number of pairs in each category: SL∩!FL, !SL∩FL, and SL∩FL, respectively.

Among a total of 456 neuron–muscle pairs, 266 pairs showed either a SL (PSF or PSS) or a FL (positive or negative), and 43 pairs concurrently showed both SL and FL (SL∩FL, Figure 3A, Table 2). The existence of SL and FL was significantly associated (*p* = 0.014, χ^2^ = 6.0); a greater proportion of pairs with significant SL than pairs without significant SL also showed significant FL (43/63 pairs, 68%, and 203/393, 52%, respectively), and only 20/63 pairs with significant SL lacked the significant FL. This clear association between SL and FL suggested that spinal PreM-IN output effects significantly modulate target muscle activities.

**Table 2:** **Associations between SL and FL**.

**Functional linkage**				
**Synaptic linkage**	**Positive**	**Negative**	**No FL**	**Total (pairs)**
**EXCITATORY PreM-INs (356 PAIRS)**				
PSF	42	0	9	51
No effect	176	15	114	305
Total	218	15	123	356
**INHIBITORY PreM-INs (100 PAIRS)**				
PSS	1	0	11	12
No effect	12	0	76	88
Total	13	0	87	100
**ALL PreM-INs (456 PAIRS)**				
PSE	43	0	20	63
No effect	188	15	190	393
Total	231	15	210	456

Interestingly, SL and FL association depended on whether the PreM-INs were excitatory or inhibitory (Figures 3B,C). In the excitatory PreM-INs, the majority (42 of 51; 82%) of neuron–muscle pairs with a significant SL (PSF) also showed a significant FL, and the association between SL and FL was significant (*p* = 0.006, χ^2^ = 7.5). On the other hand, for the inhibitory PreM-INs, only one of 11 neuron–muscle pairs with significant SL (PSS) showed a significant FL, and the association was not significant (*p* = 0.6, χ^2^ = 0.3). This result suggested that excitatory PreM-INs constituted the prime movers of the target muscle activity; inhibitory PreM-INs were involved to a lesser extent.

Although there was a significant association between SL and FL, there were many exceptions: 20 pairs had SL without FL (SL∩!FL), and 203 pairs had FL without SL (!SL∩FL). To test whether these incongruities occurred by chance, we quantified the chance level of these incidences. PSE significance was tested using *p* < 0.0025 (*P*_*SL*_), and cross-correlation significance was tested using *p* < 0.01 (*P*_*FL*_). Therefore, the chance level of SL∩!FL and !SL∩FL was set to *P*_SL_^*^ (1 − *P*_*FL*_) and (1 − *P*_*SL*_)^*^ *P*_*FL*_, respectively. A binomial test showed that the number of SL∩!FL (*n* = 20) and !SL∩FL (*n* = 203) significantly exceeded the chance level (*p* < 0.001, binomial test). These results indicated that SL and FL were clearly associated, but a significant number of incongruities between SL and FL also existed.

## Discussion

The existence of hand-muscle synergy and the modular control of primate grasping has been suggested (Brochier et al., 2004; Overduin et al., 2008), but neural implementation of hand-muscle synergy remained unclear. Here we explored how PreM-IN output effects contributed to hand-muscle activation by investigating the PSEs of PreM-INs on hand muscles (i.e., SL) and the long-term cross-correlation between PreM-IN and hand-muscle activity (i.e., FL). Our results showed that the existence of SL and FL were significantly associated and suggested that spinal PreM-IN output effects significantly contribute to hand-muscle activity modulation during grasp control. However, we also found considerable incongruities between SL and FL. This result suggested that although the PreM-IN output projections significantly affect hand-muscle activity modulation, other neural structures are needed to recruit an adequate combination of hand-muscle motoneurons.

### Association between spinal PreM-IN SL and FL with hand-muscle activity

The contribution of spinal interneurons to muscle synergy has been extensively investigated in the hind-limb movement of frogs (Giszter et al., 1993; Mussa-Ivaldi et al., 1994; Tresch et al., 1999; Saltiel et al., 2001; Bizzi et al., 2002; Hart and Giszter, 2010) and rats (Tresch and Bizzi, 1999). However, it was not self-evident that the analogous neural mechanism could be assumed for the primate cervical spinal cord and the control of hand grasping. Our results revealed that spinal PreM-INs in the primate cervical cord had divergent output effects on hand muscles and significantly functioned to modulate target muscle activity, suggesting that they could be a part of the neural implementation of hand-muscle synergy. This is analogous to frog lumbar spinal interneurons (Hart and Giszter, 2010). In hind-limb movement, a small number of motor primitives are represented in spinal cord, and their combination can construct a variety of reflexive and natural movements (d'Avella et al., 2003). As the motor primitives exist in the lower CNS (i.e., spinal cord), the control dimension in the higher motor structures might be reduced (Tresch and Jarc, 2009). Similarly, primate hand movements are characterized by very high DOF (Ogihara and Oishi, 2012) and therefore may have a computational advantage if a neural structure for hand muscle synergy is implemented in the spinal cord.

The clear association between SL and FL was specific to excitatory PreM-INs and was not found in inhibitory PreM-INs (Figures 3B,C). This suggests a functional difference between excitatory and inhibitory spinal PreM-INs related to the control of primate grasping. Excitatory PreM-INs mostly positively covaried with the target muscles (Figure 3B), suggesting that excitatory PreM-INs were a prime mover of hand-muscle coactivation. Conversely, few inhibitory PreM-INs significantly covaried with target muscles (Figure 3C). Because no inhibitory PreM-INs showed significant negative covariation with target muscle activity (Table 1), inhibitory PreM-INs may function to adjust the activities and response gains of agonist muscles (Chance et al., 2002; Berg et al., 2007; Kristan, 2007), rather than reciprocally inhibiting antagonist muscles.

### Possible mechanisms of incongruence between SL and FL

**Figure 4:**
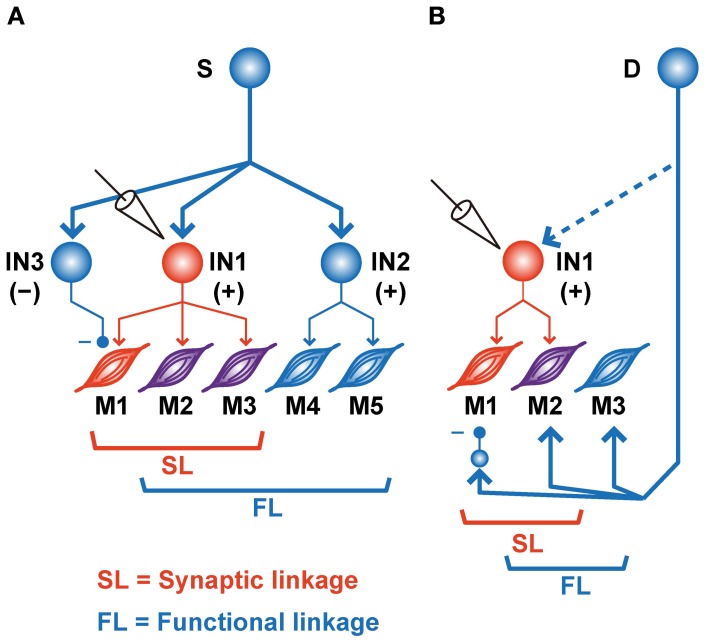
**Schematic illustrations of various SL and FL combinations. (A)** Common input (S) to the various types of PreM-INs. IN1, recorded excitatory PreM-IN; IN2 and IN3, non-recorded excitatory and inhibitory PreM-INs, respectively. EMGs are categorized as SL∩!FL (M1, *red*), SL∩FL (M2–3, *purple*), or !SL∩FL (M4–5, *blue*). **(B)** A premotor input (D) parallel to PreM-IN. IN1, recorded excitatory PreM-IN. Again, EMGs are categorized as SL∩!FL (M1, *red*), SL∩FL (M2, *purple*), or !SL∩FL (M3, *blue*).

In addition to the significant association between SL and FL, our results also showed a significant number of incongruities, i.e., !SL∩FL and SL∩!FL (Figures 2, 3). Several mechanisms could explain these incongruities (Figure 4). First, the incongruities can be explained by assuming a common input into several PreM-INs, which have different types of muscle field. Figure 4A shows a schematic illustration of how a common input (“S”) can produce these incongruities. Common input into two excitatory (IN1 and IN2) and one inhibitory PreM-IN (IN3) induces synchronization among these PreM-INs. This synchronization, in turn, would induce covariation between the activity of the recorded PreM-IN (IN1) and its non-target muscles (M4–5) due to the synchronized excitatory PreM-IN (IN2) input to them (!SL∩FL). Additionally, the inhibitory PreM-IN (IN3), synchronized with the recorded PreM-IN, suppresses the shared target muscle activity (M1), and this might result in decorrelation between IN1 and M1, even though IN1 had a synaptic effect on M1 (SL∩!FL). The correlation between spinal INs reported by Prut and Perlmutter (2003) may have been induced by divergent branching of the descending (Shinoda et al., 1981; Li and Martin, 2002) and afferent (Ishizuka et al., 1979; Brown, 1981; Ralston et al., 1984) axons to the spinal cord.

Another possible explanation for the incongruities is the involvement of other premotor systems (Figure 4B). If a premotor system (“D”), parallel to the spinal PreM-INs, primarily contributes to the formation of the hand-muscle coactivation pattern, the SL and FL between PreM-IN and the target muscle could produce incongruities (Figure 4B). For example, if a premotor system coactivates the target muscles (M2–3) and the recorded PreM-IN (IN1) while suppressing some of the target muscles (M1) via inhibitory neurons, the incongruences will occur between SL and FL of the recorded PreM-IN and its target muscles (M1 is SL∩!FL and M3 is SL∩!FL). Every premotor system that bypasses spinal PreM-INs [e.g., corticomotoneuronal (CM), rubromotoneuronal (RbM), reticulomotoneuronal (RtM), and group-Ia primary afferent cells] is a possible candidate for the premotor system that contributes to the coactivation of hand-muscle activity. CM cells have a selective hand-muscle field (Buys et al., 1986), and they could function to coactivate a small group of hand muscles. However, CM neurons are specifically active during precision grip as compared with power grip (Muir and Lemon, 1983), and their firing increases when one of the their target muscles is more active than another, in contrast to equal coactivation of the target muscles (Bennett and Lemon, 1996). Therefore, CM cells might function to fractionate hand-muscle activity rather than simply to coactivate the target muscles. The relative contribution of CM cells and PreM-INs to hand-muscle activity control and hand-muscle synergies should be further tested. RbM cells are another candidate for constructing muscle synergy. Several studies showed that RbM cells have a divergent hand-muscle field (Mewes and Cheney, 1991; Sinkjaer et al., 1995). However, it has been reported that their muscle field is strongly biased toward the forearm extensors (Mewes and Cheney, 1991; Sinkjaer et al., 1995); cells in the magnocellular division of the red nucleus, where most RbM cells are located, are preferentially activated when monkeys preshape their hand rather than when they grasp objects during a reaching-to-grasp task (Van Kan and McCurdy, 2001, 2002). These results suggested that RbM cells mainly contribute to constructing the muscle synergy for preshaping the hand rather than for grasping objects. Finally, Davidson (2011) recently reported the PSEs of pontomedullary reticular formation (PMRF) neurons on the extrinsic hand muscles (Davidson, 2011); hence, RtM cells may function to modulate hand-muscle activity involved in the control of grasping. In addition to these descending sources, Ia afferent to spinal motoneurons, which are also obvious premotor neurons, show task-relevant activity during wrist movement (Flament et al., 1992), but their contribution to hand-muscle movement is unknown. The afferent feedback may include functions that modulate the SL–FL relationship and define the final muscle activities according to context and external event. So far, as seen in these previous reports, the contributions of each descending tract to hand grasping have been separately investigated. Therefore, the differential contributions of these multiple premotor systems (spinal PreM-INs, CM, RbM cells, RtM cells, and primary afferents) to the control of hand grasping and the mechanism of their coordination for control of hand grasping remain to be clarified. To approach this issue, it is crucial to directly compare functional differences in the contributions of these parallel premotor systems to the formation of hand muscle synergy under the same behavioral paradigm and in the same subjects.

## Conclusions and future directions

In this study, we explored how PreM-IN output effects contribute to hand-muscle activation by investigating SL and FL between PreM-INs and hand-muscle activity in monkeys performing a precision grip task. Our results showed that SL and FL between PreM-IN and their target muscle activities were significantly associated, indicating that spinal PreM-INs contribute to hand-muscle activity modulation during control of grasping. However, a significant number of incongruities between SL and FL were also found, suggesting the contribution of other neural structures in recruiting an adequate combination of hand-muscle motoneurons. Further studies are needed to elucidate the relative importance of multiple premotor systems to the control of hand-muscle activity during grasping.

The co-existence of associations and incongruities between SL and FL may reflect that the modular control of hand movements is characterized by both fixed and flexible control (Macpherson, 1991). First, the clear association between SL and FL indicates that synaptic connections from PreM-INs significantly contribute to the modulation of hand-muscle activity. As PreM-INs have a divergent muscle field, these neuroanatomical or hardwired connections may produce the invariant activation patterns of the hand-muscle activities. On the other hand, the fact that FL is not always restricted to instances of SL but can be dissociated from the latter suggests that FL may be flexible according to the context or tasks (Nazarpour et al., 2012). Let us imagine that the monkeys in this study performed a different type of grasping task (e.g., power-grip task) in addition to the precision-grip task. It is possible that the PreM-INs activated during the precision grip would also be recruited in a different grasping task and that the mechanism shaping FLs would be flexible enough to modify the basic pattern of the SLs according to task demands. In this case, the SL for a specific movement would be generalizable to other types of movement. Alternatively, it is also possible that the power-grip task would recruit populations of PreM-INs, producing SLs that differed from those recruited for the precision grip, and that the PreM-INs would form FLs that would be adequate for the power-grip task. In this case, the generalization of a given SL would be rather limited, and a different movement would be controlled by different PreM-INs that exhibit unique SLs. Although results in this paper suggest a flexible FL, these two possibilities may not be mutually exclusive. Further studies investigating PreM-IN firing during different types of grasping may contribute to understanding the invariance and flexibility of the modular control of hand movements.

### Conflict of interest statement

The authors declare that the research was conducted in the absence of any commercial or financial relationships that could be construed as a potential conflict of interest.
